# Lessons learnt from human papillomavirus (HPV) vaccination in 45 low- and middle-income countries

**DOI:** 10.1371/journal.pone.0177773

**Published:** 2017-06-02

**Authors:** Katherine E. Gallagher, Natasha Howard, Severin Kabakama, Sandra Mounier-Jack, Ulla K. Griffiths, Marta Feletto, Helen E. D. Burchett, D. Scott LaMontagne, Deborah Watson-Jones

**Affiliations:** 1 Clinical Research Department, London School of Hygiene and Tropical Medicine, London, United Kingdom; 2 Mwanza Intervention Trials Unit, National Institute for Medical Research, Mwanza, Tanzania; 3 Department of Global Health and Development, London School of Hygiene and Tropical Medicine, London, United Kingdom; 4 PATH, Center for Vaccine Innovation and Access, Seattle, Washington, United States of America; Banaras Hindu University, INDIA

## Abstract

**Objective:**

To synthesise lessons learnt and determinants of success from human papillomavirus (HPV) vaccine demonstration projects and national programmes in low- and middle-income countries (LAMICs).

**Methods:**

Interviews were conducted with 56 key informants. A systematic literature review identified 2936 abstracts from five databases; after screening 61 full texts were included. Unpublished literature, including evaluation reports, was solicited from country representatives; 188 documents were received. A data extraction tool and interview topic guide outlining key areas of inquiry were informed by World Health Organization guidelines for new vaccine introduction. Results were synthesised thematically.

**Results:**

Data were analysed from 12 national programmes and 66 demonstration projects in 46 countries. Among demonstration projects, 30 were supported by the GARDASIL^®^ Access Program, 20 by Gavi, four by PATH and 12 by other means. School-based vaccine delivery supplemented with health facility-based delivery for out-of-school girls attained high coverage. There were limited data on facility-only strategies and little evaluation of strategies to reach out-of-school girls. Early engagement of teachers as partners in social mobilisation, consent, vaccination day coordination, follow-up of non-completers and adverse events was considered invaluable. Micro-planning using school/ facility registers most effectively enumerated target populations; other estimates proved inaccurate, leading to vaccine under- or over-estimation. Refresher training on adverse events and safe injection procedures was usually necessary.

**Conclusion:**

Considerable experience in HPV vaccine delivery in LAMICs is available. Lessons are generally consistent across countries and dissemination of these could improve HPV vaccine introduction.

## Introduction

Globally, an estimated 528,000 new cervical cancer cases and 266,000 deaths occur annually [1]. Over 85% of new cervical cancer cases occur in women living in low and middle-income countries (LAMICs), who have limited access to screening services [1–4]. There are three licensed prophylactic HPV vaccines against persistent infection with HPV vaccine genotypes and high-grade cervical intraepithelial neoplasia, pre-requisites for cervical cancer development [5]. Cervarix^®^ (GlaxoSmithKline Biologicals) targets HPV genotypes 16 and 18; GARDASIL^®^ (Merck & Co. Inc.) targets HPV 16, 18, 6, 11 [6]; GARDASIL-9 (Merck & Co. Inc.) targets an additional five oncogenic genotypes [7]. As HPV is sexually transmitted, the World Health Organization (WHO) recommends targeting HPV vaccination to girls prior to sexual debut (e.g. age 9–13) because it is most efficacious in those who have not been exposed to HPV [8].

Between 2007 and 2012, several LAMICs conducted HPV demonstration projects with vaccines provided by the GARDASIL^®^ Access Program (GAP) [9], Merck & Co., the Bill & Melinda Gates Foundation through PATH, or through other means. Demonstration projects are small-scale pilots through which experience can be gained in delivering the vaccine to what is often a novel target age group [10]. In 2012 Gavi, the Vaccine Alliance, commenced support for demonstration projects and national introductions to increase access to HPV vaccine worldwide. The majority of demonstration projects are now Gavi-funded. National programmes may also be funded by Gavi if the country has prior experience of vaccination in the target age group and achieved over 50% vaccination coverage. By May 2016, over 80 countries or territories had commenced national HPV vaccination and another 38 had completed or started HPV vaccine demonstration projects [11].

Country decision-makers face several challenges when applying for support and introducing HPV vaccine including selection of delivery strategy, effective communication with communities and determining how to maximise coverage [12, 13]. At the time of this study, no comprehensive review of results and lessons learnt from demonstration projects or early scale-up in LAMICs had been conducted. This study aimed to synthesise lessons learnt from the HPV demonstration projects and national programmes in LAMICs implemented between January 2007 and May 2016 to develop recommendations for HPV vaccine delivery and accelerate scale-up of national programmes.

## Methods

This ecological study included semi-structured key informant interviews, a systematic literature review and a review of unpublished reports. Units of analysis were: 1) countries, 2) projects/programmes, and 3) delivery experiences (Table 1).

**Table 1:** Key definitions.

Delivery experience	The specific target population (age range in years or school grade) and vaccination venue (health facility-based, school-based, outreach, or a combination of the three) within a specific project/programme (defined by the funding source). E.g. A country that was funded for 2 years for a demonstration project and implemented one year of school-based delivery and a second year of health facility based strategy, was classified as having contributed information from one project but two delivery experiences.
Programme	A national HPV vaccination programme.
Project	The activities funded through a specific GAP, Gavi or other funder support for a demonstration/pilot project. A distinct project was defined by the funder and/or implementer and grant award details.

A mapping exercise identified all low and lower-middle income countries that had completed at least six months of an HPV vaccine demonstration project or national programme by the end of April 2016. Data from upper-middle or high-income countries were only included if they conducted a demonstration project or utilised an innovative dosing schedule (n = 46; Table 2) [14]. At least another 6 LAMICs were planning to start Gavi-supported demonstration projects, but did not have data in time for inclusion in this study.

**Table 2:** Countries included in this study with publications included from the systematic literature search.

Country	Income^1^	Primary school net enrolment ratio^2^	Demo^3^/ National (funding source)^3^	Vaccination venue(s)	Year/s HPV vaccination
**Bhutan** [15–21]	Lower-middle	88.1 (2013)	Demo (GAP)	School	2009
			National (ACCF)	School	2010
				Health facility + outreach	2011–13
				School + health facility + outreach	2014-
**Bolivia** [19, 20]	Lower-middle	81.6 (2013)	Demo 1 (GAP)	School + health facility	2009
			Demo 2 (GAP)	School + health facility + outreach	2009
			Demo 3 (GAP)	School + health facility	2010
			Demo 4 (GAP)	School + health facility	2010–11
**Botswana** [22–24]	Upper-middle	83.8 (2009)	Demo (WB)	School	2013
			Demo (MOH)	School + health facility	2014
			National (Govt.)	School + health facility	2015
**Brazil** [25–29]	Lower-middle	94.4 (2005)	Demo (GAP)	School	2010–11
			Demo (MOH)	School + outreach	2010–12
			National (Govt.)	School + health facility	2014-
**Burkina Faso**	Low	67.5 (2013)	Demo (Gavi)	School + health facility + outreach	2015-
**Cambodia** [19, 20]	Low	98.4 (2012)	Demo 1 (GAP)	Health facility	2009–10
			Demo 2 (GAP)	School + health facility	2010–11
**Cameroon** [19, 20, 30–34]	Lower-middle	91.5 (2012)	Demo 1 (GAP)	School + health facility	2010
			Demo 2 Gavi)	School + health facility + outreach	2015-
**Chile** [35]	High	92.7 (2012)	National (Govt.)	School + health facility	2014-
**Côte d’Ivoire**	Lower-middle	61.9 (2009)	Demo (Gavi)	School + health facility + outreach	2015-
**Ethiopia**	Low	67.9 (2006)	Demo (Gavi)	School + outreach	2015-
**The Gambia**	Low	68.7 (2013)	Demo (Gavi)	School + health facility + outreach	2015-
**Georgia** [20]	Lower-middle	96.5 (2013)	Demo 1 (GAP)	Health facility	2010
			Demo 2 (GAP)	Health facility + outreach	2010–14
**Ghana**	Lower-middle	88.9 (2014)	Demo 1 (GAP)	School	2013
			Demo 2 (Gavi)	Year 1: School. Year 2: School + health facility + outreach	2013–15
**Guyana**	Lower-middle	71.5 (2012)	Demo (GAP)	School + health facility	2012–13
			National (Govt)	NA	2014
**Haiti** [19, 20]	Low	NA	Demo (GAP)	School	2009
**Honduras** [20]	Lower-middle	89.3 (2013)	Demo 1 (GAP)	School + health facility + outreach	2011
			Demo 2 (GAP)	School	2012–13
			Demo 3 (GAP)	School + health facility	2014
			National (Govt.)	School + health facility	2015-
**India** [36–40]	Lower-middle	93.3 (2011)	Demo (PATH)	School + health facility campaign	2009–10
				School and health facility monthly delivery	2009–10
**Kenya** [20, 41, 42]	Low	83.6 (2012)	Demo (GAP)	School	2011
			Demo (Gavi)	School	2013–15
**Kiribati**	Lower-middle	NA	Demo (GAP/ ACCF)	School	2011–13
**Laos PDR**	Lower-middle	97.3 (2013)	Demo (Gavi)	School + health facility + outreach	2013–15
**Lesotho** [19, 20]	Lower-middle	79.6 (2013)	Demo 1 (GAP)	School	2009
			Demo 2 (GAP)	School	2010–11
			National	School	2012-
**Madagascar**	Low	77.1 (2003)	Demo (Gavi)	School + health facility	2013–15
**Malawi**	Low	96.9 (2009)	Demo (Gavi)	School + health facility	2013–15
**Mali**	Low	64.4 (2013)	Demo 1 (GAP)	Health facility	2012
			Demo 2 (Gavi)	School + health facility + outreach	2015-
**Moldova** [20]	Lower-middle	87.9 (2013)	Demo (GAP)	School	2010–11
**Mongolia**	Lower-middle	94.7 (2013)	Demo (GAP)	School + health facility + outreach	2012
				School	2014
**Mozambique**	Low	87.4 (2013)	Demo (Gavi)	School + health facility + outreach	2014–15
**Nepal** [19, 20, 43]	Low	98.5 (2013)	Demo 1 (ACCF)	School	2008
			Demo 2 (GAP/ACCF)	School + health facility	2010
			Demo 3 (ACCF)	School + health facility	2011–14
			Demo 4 (Gavi)	School + health facility	2015-
**Niger**	Low	62.8 (2012)	Demo (Gavi)	School + outreach	2014–15
**Papua New Guinea**	Lower-middle	85.6 (2012)	Demo (GAP)	School + health facility	2012
**Peru** [37–39, 44–49]	Upper-middle	91.8 (2013)	Demo (PATH)	School + health facility + outreach	2007–08
					2009–10
			National (Govt)	School	2011–12 2014-
**Philippines**	Lower-middle	88.2 (2009)	Demo (Jhpiego)	NA	2010
**Rwanda** [50–53]	Low	93.4 (2013)	National (Merck)	School + health facility + outreach	2011–13
			National (Gavi)	School + health facility	2014-
**Senegal**	Lower-middle	73.4 (2014)	Demo (Gavi)	School + health facility + outreach	2015-
**Sierra Leone**	Low	NA	Demo (Gavi)	NA	2013
**Solomon Islands**	Lower-middle	80.7 (2007)	Demo (Gavi)	School + health facility + outreach	2015-
**South Africa** [54–61]	Upper-middle	89.6 (2005)	Demo 1 (UCT)	Health facility	2010
			Demo 2 (KZN DoH)	School	2011
			Demo 3 (UoS)	School	2013
			National (Govt.)	School	2014-
**Tanzania** [20, 39, 62–68]	Low	83.5 (2013)	Demo 1 (GAP)	School—age and grade criteria tested	2010–11
					2010–11
			Demo 2 (Gavi)	Year 1: School & health facility. Year 2: Health facility + outreach	2014-
					*2016-*
**Thailand** [69, 70]	Upper-middle	95.6 (2009)	Demo (Jhpiego)	NA	2010
**Togo**	Low	97.5 (2013)	Demo (Gavi)	School + health facility + outreach	2015-
**Uganda** [20, 37–39, 48, 71–81]	Low	91.5 (2013)	Demo 1 (PATH/ MOH)	School + health facility	2008–09 2010–11
				School + health facility + outreach	2008–09 2010–11
			Demo 2 (GAP)	Health facility	2010
			Demo 3 (Merck)	School + outreach	2012–14
			Natl (Gavi)	Health facility + outreach	2015-
**Uzbekistan** [20]	Lower-middle	88.5 (2011)	Demo (GAP)	Health facility	2009
			*National (Gavi)*	*School + health facility*	*2016-*
**Vanuatu**	Lower-middle	98.9 (2005)	Demo (ACCF)	School	2009
			National (ACCF)	School + outreach	2013-
**Vietnam** [37–39, 48, 72, 79, 82–84]	Lower-middle	98.1 (2012)	Demo (PATH/ MOH)	School + health facility	2008–10
				Health facility	2008–10
**Zambia**	Lower-middle	91.4 (2013)	Demo (GAP)	School + health facility	2013–14
**Zimbabwe**	Low	93.9 (2012)	Demo (Gavi)	School + health facility + outreach	2015-

^1^ World bank classifications of income group, February 2014.

^2^ Information sourced from UNESCO Institute of Statistics, educational attainment most recently available data; year is indicated in brackets.

*Italicised text* indicates experiences with incomplete data due to start date; this data was obtained in the process of data collection when countries were questioned about future or current HPV vaccine activity; only experiences with at least one year of implementation were included in analyses.

**Abbreviations:** ACCF, Australian Cervical Cancer Foundation; CHW, community health worker; Demo, demonstration/pilot project; GAP, Gardasil^®^ Access Program; est., estimated; HPV, human papillomavirus; KZN DoH, KwaZulu-Natal Department of Health; MOH, ministry of health; national, national programme; NA, not available; UCT, University of Cape Town; UNESCO, the United Nations Educational Scientific and Cultural Organisation; UoS, University of Stellenbosch; WB, World Bank.

### Systematic literature review

**Fig 1:**
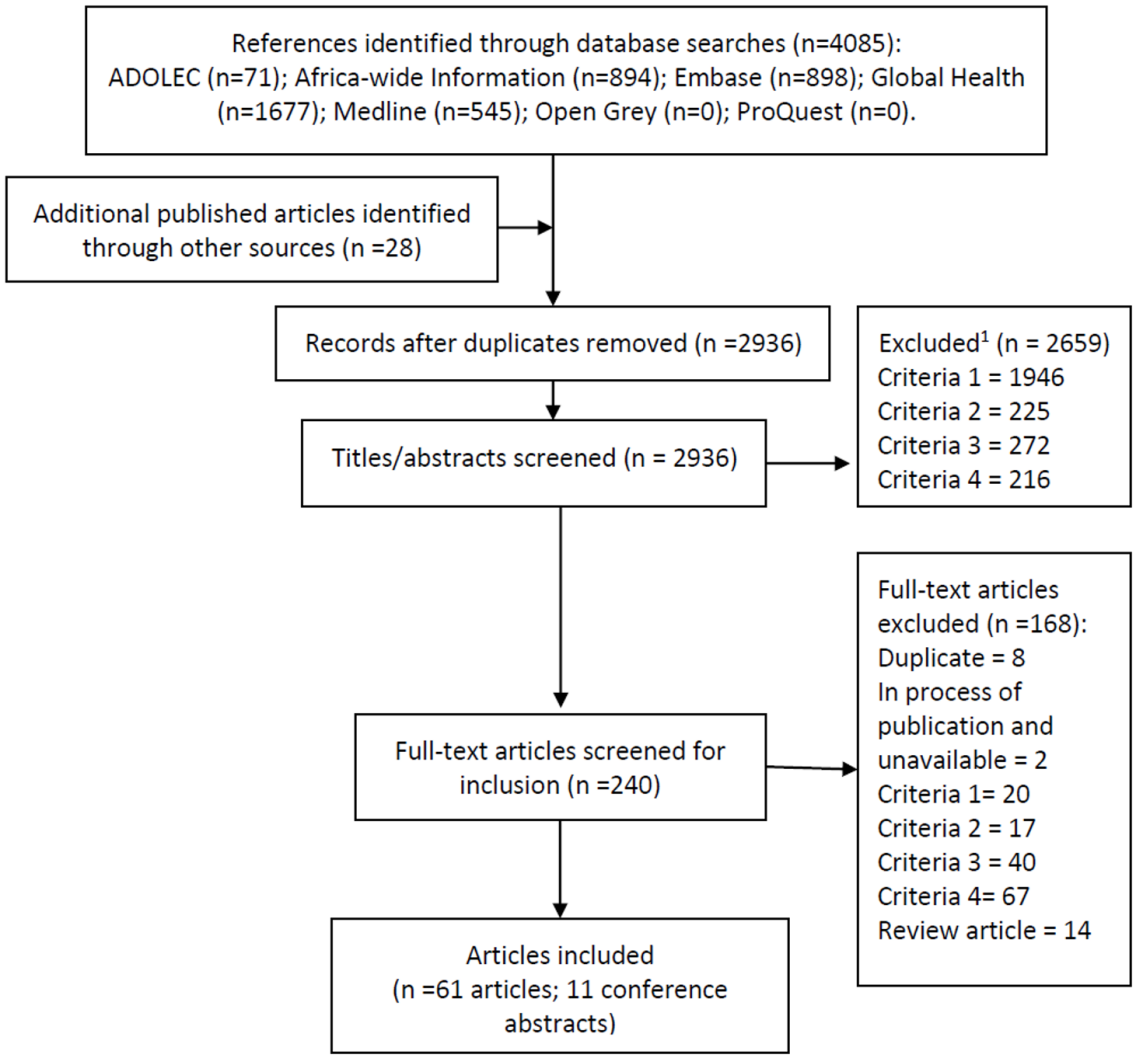
Systematic literature review flow. ^1^Exclusion criteria were: 1) not focused on HPV vaccination; 2) not focused on one of our countries of interest; 3) did not include any results from after the vaccine was delivered; 4) not focused on, or relevant to, the demonstration project or vaccine introduction. Review articles were identified and searched for further references but were not included in data extraction.

Five databases (Medline, Embase, Global Health, Africa-wide Information, ADOLEC) were searched systematically for published literature in April 2016. Search terms relating to HPV and vaccination were combined with country terms, with no language restrictions (S1 Table). For each country, searches were limited to publications from the first year of HPV vaccine experience onwards, to reduce the number of articles retrieved that did not document vaccine delivery (e.g. hypothetical acceptance studies). Reference lists of identified reviews and retrieved papers were checked for missing papers. One author was contacted for an unpublished manuscript. Titles and abstracts of 2936 references were double screened by two of three study investigators using exclusion criteria set *a-priori* in a protocol as per PRISMA guidelines [85] (Fig 1). Exclusion criteria were: 1) not focused on HPV vaccination; 2) not focused on one of our countries of interest; 3) did not include any results from after the vaccine was delivered; 4) not focused on, or relevant to, the demonstration project or vaccine introduction. Any conflicting opinions between investigators on the exclusion of abstracts were noted and resolved after full text review by the third investigator. A total of 240 full texts were screened by the same study personnel using the same exclusion criteria. Review articles were identified and searched for further references but were not included in the final selection of articles for data extraction.

### Unpublished reports

Authors systematically searched two databases (Open Grey, ProQuest) and several websites (national Ministries of Health (MOH), WHO Global Immunization News, Pan-American Health Organization newsletters, scientific conferences on HPV) for unpublished literature through March 2016. Unpublished data were solicited directly from country representatives and stakeholders involved in HPV projects/programmes.

### Key informant interviews

Representatives from each HPV vaccine project/programme in 44 of the 46 countries were approached for interview in order to fill gaps in the data in the published and unpublished literature. No significant gaps were identified in two countries. After written informed consent was obtained, interviews were conducted by phone or in-person. A topic guide was adapted to address identified knowledge gaps. All interviewees were assured of confidentiality and anonymity to encourage openness about experiences.

### Data extraction

KG, NH, SK, SMJ extracted data during February-May 2016, using an Excel-based matrix of key areas of inquiry informed by WHO’s new vaccine introduction guidelines [86]. The matrix was piloted and revised twice, with two consistency checks conducted. Data from published, unpublished and interview sources were extracted into the same matrix.

### Data analysis

Country data from all sources were triangulated and analysed together in seven themes: preparation, communications, delivery, achievements, sustainability, integration and value of demonstration projects. Data were grouped by calendar year, world region and type of funder or implementer to analyse patterns.

Qualitative data were analysed thematically across data sources. Quantitative data (e.g. coverage, adverse events) were analysed descriptively to present frequencies and proportions. Reported coverage estimates were categorised as percentages because not all projects/programmes shared numerator and denominator data to enable coverage calculations.

The London School of Hygiene & Tropical Medicine Research Ethics Committee approved the study in March 2015.

## Results

In total, 61 published articles, 11 conference abstracts, and 188 unpublished documents were included in the review. Unpublished documents received from country representatives and international partners from 44 of the 46 countries included: GAP final reports (n = 16); Gavi post-introduction evaluations (PIEs; n = 9); other PIEs (n = 2); Gavi cost analyses (n = 6); Gavi coverage surveys (n = 9); and other internal reports (n = 146). Additionally, 56 interviews were conducted covering experiences from 40 countries (59 demonstration projects, 11 national programmes). Four country representatives invited to interview either refused to participate or did not respond.

The 46 countries that implemented HPV vaccination projects/programmes between January 2007 and May 2016 accumulated 120 years of implementation experience (Table 2). This included 12 national programmes and 66 demonstration projects. By May 2016, 39% of countries (n = 18) had 2–3 years of experience, 35% (n = 16) had one year of experience and 26% (n = 12) had four or more years of experience in national programmes or multiple demonstration projects. Twenty-one projects/programmes in 19 countries had implemented a two-dose HPV vaccine schedule by May 2016; all others implemented a three-dose schedule. HPV vaccination was free-of-charge to recipients in all projects/programmes.

### Preparation

#### Leadership and planning

Three-quarters of projects/programmes were led by the MOH, with lead departments varying between cancer, school/sexual/reproductive health and immunisation. Some early demonstration projects were led by hospitals or non-governmental organisations (NGOs) with varying degrees of national immunisation team (EPI) involvement, a few operated without government input. Some interviewees from countries without school/adolescent health programmes reported confusion over which department should lead coordination of HPV vaccination and leadership was decided opportunistically, based on capacity. However, it was clear that EPI involvement was necessary to ensure smooth implementation and reduce workload (e.g. to avoid establishment of parallel vaccine management and reporting systems). Delivery experiences with MOH ownership and high EPI involvement were more likely to achieve good coverage in comparison to others run by external partners or with low EPI involvement. Sources indicated that to be effective, microplanning needed involvement of the Ministry of Education (MOE), teachers and school administrators and health representatives.

#### District selection

Among the 53 projects in 40 countries with data, areas included in a quarter of the demonstration projects represented those with routine immunisation coverage and education performance similar to the national average (15 projects), a fifth represented convenient districts (i.e. close to the capital city and/or had good infrastructure; 10 projects), 30% were representative of both urban and rural areas (16 projects). Projects could be classified in more than one of these categories. Some projects selected districts that included varied or particularly challenging areas (13%, 7 projects) but 17% (5 projects) selected areas with higher than national average EPI coverage and educational attainment.

#### Enumeration of the population eligible for vaccination

Accurate enumeration was challenging in most countries and affected estimations of the doses required, transport and coverage calculations. School headcounts/ register checks used in conjunction with school enrolment rates, were the most accurate methods to calculate the target population number, aside from conducting a full census, which was prohibitively expensive in most countries. However, the number of out-of-school girls was often unknown throughout projects/ programmes.

#### Cold-chain and waste management

The most efficient method of transporting HPV vaccines was alongside other routine vaccines. However, in some countries this proved problematic due to the demonstration project timeline not aligning with quarterly vaccine delivery schedules. Providing separate transport increased the cost of delivery. Routine national immunization cold-chain facilities were generally used. Waste management generally followed routine practices and needed improvement in many countries.

#### Staff training

Cascade training (i.e. national staff training regional staff, who train district-level, who train field-staff), was reportedly less expensive than transporting teams of national trainers around the country. However, periodic supervision was considered necessary in order to ensure that the quality of information transfer between levels in the cascade was maintained.

### Communications

HPV vaccination as a cancer prevention method was more frequently emphasized than its role in sexually transmitted infection (STI) prevention, in order to avoid stigmatising the vaccine and to reduce confusion with other STI prevention messages [87]. Messages targeted the whole community with information focused on cervical cancer, the importance of HPV vaccination, government endorsement, doses required, timing and venues, and lack of long-term adverse effects. Problems were reported when social mobilisation occurred less than a month before vaccination and high-level officials did not deal with rumours rapidly.

### Delivery

#### Venue and target

Schools were the most commonly used vaccination venue, with 87% (78/89) of delivery strategies using them, with or without additional health facility or outreach components (Table 2). Strategies including schools gained high coverage but were reported to be resource intensive in countries without existing school-based health programmes. There were limited data on strategies that used health facilities as the only sites of vaccine delivery (11 experiences, 5 with coverage data). In experiences that used schools, 52% (39/75) vaccinated a specific age group of girls, 31% (23/75) selected a school grade(s) and 17% (13/75) vaccinated girls of a certain age within a specific school grade. Some MOH-led projects/ programmes made changes to the delivery strategy for a variety of reasons that illustrate the trade-offs inherent in different strategies (Table 3).

**Table 3:** Changes in delivery strategy.

**Countries**	**Original strategy**	**Change in strategy**	**Reasons for changes**
Change from school-based campaign (3 countries)	School or school + health facility	Health facility with/ without outreach	High level of resources required for outreach visits to schools and concern over sustainability. One country subsequently switched back to school based strategy, as HPV vaccine coverage was low with health facility delivery.
Removal of out-of-school strategy (2 countries)	School + health facility + outreach	School + health facility or school-only	Outreach had proven resource intensive, with logistical difficulties and only incremental gains in coverage.
Addition of strategies to reach out-of-school girls (5 countries)	School or Health facility	School + health facility +/- outreach or Health facility + outreach	To increase coverage and equity of HPV vaccination by including out-of-school girls.
**Countries**	**Original target population**	**Change to target population**	**Reasons for changes**
Change to identification of girls by grade (5 countries)	Age	Grade	Identifying eligible girls by age was difficult if exact birth date/year was not known or documented. It was unacceptable to separate some girls from their classmates to receive the vaccine while other class members were not vaccinated.
Change to identification of girls by age (4 countries)	Grade or age within a grade	Age	It is easier to explain to the community and aligns with routine EPI, which used age cohorts. Easier to estimate the denominator/ target population even if girls are spread in different grades. To purposely assess a different strategy in the second year of the project.
Adaption of age/grade criterion to be more appropriate (5 countries)	Grade	More appropriate grade	A higher concentration of eligible girls were in a higher/lower grade.
	Age 10 out-of-school	Age 9–13 out-of-school	The relative ease of identifying ‘pre-pubertal’ girls around the age of 9–13 years in the community in comparison to trying to find exactly age 10 girls.

#### Out-of-school girls

National primary school enrolment ratios indicate the proportion of girls out-of-school was 5% or less in 23% of the countries with data (10/43), between 6% and 20% in 56% of countries (24/43), and over 20% in nine countries (range 23–38%)[14]. Almost a third of experiences (27%) had no reported strategy for reaching out-of-school girls, another third (35%) relied on them attending health facilities for vaccination and the remaining experiences used outreach. Outreach was used in all nine countries with poor school enrolment and reportedly increased coverage.

#### Duration of delivery per dose

Duration of delivery activities per dose ranged from 2–3 days to 1 month (data from 31 delivery experiences). Most experiences delivered each dose over the course of one week and activity was synchronized across districts (i.e. similar to a vaccination campaign). Two further experiences delivered each dose over 6 months at the health facility and during routine outreach. There was no obvious relationship between the duration of delivery activities per dose and vaccination coverage. However, countries reported that it was useful to provide a second opportunity for girls to obtain the vaccine (e.g. ‘mop-up’ vaccination days at schools) if they had initially refused or were absent. The dose schedule recommendation change in April 2014 [88, 89] resulted in data on two-dose schedules from 19 countries. The delivery of two doses rather than three doses was reported as logistically easier to fit in to the school year and cheaper by all 10 countries that *had changed* vaccine schedule. One country reported an extended interval of 12 months between doses made enumeration and delivery in a single campaign each year easier.

#### Catch-up

Three national programmes conducted catch-up vaccination in older age groups either by vaccinating girls aged 9–15 or 9–18 years, or by additionally vaccinating the second and third grades of secondary school. Two further national programmes vaccinated 9–13 year olds in a small catch-up campaign in their first year of the programme. No evaluation results were available for catch-up campaigns.

#### Health workforce

Almost all countries used qualified nurses to deliver the vaccine; one used community health workers (CHWs). CHWs and teachers were reportedly invaluable at vaccination venues to ensure efficient delivery. Disruption of other health services during HPV vaccine delivery was not homogenous within a country. Strategies to minimise the impact of the HPV school/outreach activities on routine services included: integration into existing outreach days, longer working days, use of staff from other areas or services and task-shifting responsibilities to CHWs. One country delivered each dose over a month instead of short ‘campaign-style’ delivery. Supervision was reported as necessary, but supervisor and vaccinator allowances and transport were frequently reported as being drivers of high delivery costs.

#### Adverse events

Reported adverse events (AEs) were below 1% and minor across 56 delivery strategies in 44 countries that provided data. Monitoring, reporting and response procedures were consistent with those for other vaccines, although teachers were mentioned as a useful and, in some countries, novel resource in monitoring AEs.

### Achievements: Vaccine uptake, completion and coverage

Coverage was reported by 65% of experiences (60/92); only 17 projects conducted coverage surveys, the remainder relied on administrative coverage. Uptake, completion and final dose coverage achievements were high, with no estimates below 50% (Table 4). Experiences that achieved high coverage included schools as a vaccination venue, had high EPI and MOE involvement in both planning and implementation and included a strategy to reach out-of-school girls if school enrolment rates were variable. Other factors reported to encourage high coverage were: political commitment, good social mobilisation, community engagement and timely delivery of the vaccine on scheduled dates within one school year.

**Table 4:** Coverage achievements across delivery experiences.

Characteristic	Uptake (number (%))^1^				Completion (number (%))				Final dose coverage^2^ (number (%))			
	≥90%	70–89%	50–69%	Total	≥90%	70–89%	50–69%	Total	≥90%	70–89%	50–69%	Total
**School only**	9 (50)	7 (39)	2 (11)	18	13 (68)	6 (32)	0	19	8 (40)	11 (55)	1 (5)	20
**Health facility (+/- outreach)**	3(60)	2 (40)	0	5	1 (20)	4 (80)	0	5	2 (40)	1 (20)	2 (40)	5
**School + health facility (+/- outreach)**	19 (58)	14 (42)	0	33	17 (57)	13 (43)	0	30	15 (43)	13 (37)	7 (20)	35
**All experiences**	31 (55)	23 (41)	2 (4)	56	31 (57)	23 (43)	0	48	25 (42)	25 (42)	10 (17)	60

^1^ Counts of the number of experiences achieving each category of coverage are presented with row percentages, i.e. among those strategies with data, 57% of school only strategies obtained #x2265;90% uptake compared to 50% of health facility strategies obtaining ≥90% uptake. Excludes projects/programmes that started in 2015 or later

^2^ Coverage of a 2 or 3 dose regimen (only 10 experiences had coverage data on 2-dose regimen)

### Integration

Projects implemented with MOH involvement generally used EPI structures and processes for vaccine delivery. However, the small scale of projects made integration difficult to assess and sometimes led to establishment of parallel processes for monitoring and evaluation, supervision, vaccine transport and staff remuneration, as HPV vaccine was not seen as part of the ‘routine’ workload.

Joint delivery of HPV vaccine with other interventions was limited. One programme delivered HPV vaccine alongside a hepatitis B vaccination campaign. Eight projects/programmes attempted delivery with tetanus toxoid vaccine or deworming and vitamin A supplementation within school health programmes; six reported coverage estimates, which were variable. Educational messages on reproductive health or hygiene issues were delivered at the same time as HPV vaccine in eleven projects/programmes. Two externally-led projects/programmes delivered the first dose alongside a cervical cancer screening programme for mothers. No critical evaluations of joint delivery were available.

### Financing and sustainability

Thirty of the 66 demonstration projects were financed by GAP, through Axios Healthcare Development. GAP donated vaccine, but no delivery costs. Gavi funded 20 demonstration projects and provided vaccine and some delivery costs. For the first year of implementation Gavi provided either US$ 4.80 per girl or US$50,000 for delivery, whichever amount was largest. In the second year, funding was halved to account for start-up costs. PATH, through funding from the Bill and Melinda Gates Foundation and donated vaccine from GSK and Merck, financed four projects; 12 were supported by other means. Gavi, Merck, the Australian Cervical Cancer Foundation (ACCF) and national governments funded the national programmes.

Countries reported considerable uncertainty over the availability of future financing. The cost of school-based delivery was of concern for many where there were not existing school-based health programmes. In addition to the delivery strategy changes in Table 3, six countries stated that they planned to change from a school-based strategy to a health facility-based strategy in the future, due to the high level of resources required for school visits, specifically for transport and staff per diems.

## Discussion

There is now considerable experience in HPV vaccine delivery in LAMICs. School-based delivery to this target group is no longer ‘novel’. Many lessons have been learnt that should make planning easier for countries still considering whether to introduce HPV vaccination. Recommendations (Table 5; S2 Table) and outputs for decision-makers are available online [90].

**Table 5:** Key recommendations.

Section	Recommendations
**Preparation**	Planning processes should include representatives from the ministries of health, education and finance.
	National immunisation programme involvement is critical for effective vaccine delivery
**Communications**	Social mobilisation in communities should begin early (at least one month before vaccination, earlier if possible).
	Messages should focus on: cervical cancer prevention; safety and efficacy, including lack of fertility impact or long-term adverse effects, government endorsement, delivery timing and venues and the need to return for a second dose
	Members of government or WHO representatives should issue responses to rumours as quickly as possible.
	Consent processes should be consistent with existing routine EPI consent policy to avoid rumours.
**Delivery**	In areas with variable school attendance, specific mobilisation of out-of-school girls and an opportunity for them to receive the vaccine should be provided.
	If resources allow, planning a two-stage delivery of each dose can be successful in reaching those girls who initially refused vaccination.
	Countries need to be aware that HIV infected girls require 3 doses and should develop specific strategies to offer them the 3-dose regimen.
	Vaccination teams can include teachers and CHWs in order to decrease the number of qualified nurses needed for vaccine delivery sessions
**Achievements**	Including a component of school-based delivery can yield high coverage, if resources allow. If school enrolment is low, a mixture of strategies could be important in order to attain good coverage.
	More evaluation of health facility only strategies is needed.
	An opportunity for girls who missed doses to receive the vaccine should be supplied, either at return visits to schools or referral to health facility or outreach sites, depending on the resources available.
**Sustainability**	More research should be conducted on scale-up experiences.
	Where feasible (e.g. in terms of funding and country experience with introducing vaccines), consider phased national implementation rather than demonstration projects
	Further exploration of sustainable funding options should be conducted and disseminated, to encourage countries to scale-up demonstration projects
**Integration**	Rigorous evaluation of combined interventions with HPV vaccine delivery is needed to assess the effect on implementation, coverage, workload and cost. Funding agencies should systematically encourage this.
	Gradual integration of processes into routine processes should be planned and formalised after the first round of vaccination is completed.
	Opportunities to initiate or strengthen existing school health programmes and/or pre-adolescent/adolescent health should be seized through on-going collaboration with partners (e.g. MOE, reproductive health departments).

Our findings are limited by the variation in data availability; some topics were rarely reported, or the data was highly variable in quality (e.g. coverage). Representatives from four countries did not respond or refused interviews. As we relied on data supplied by country representatives, the availability of data may have been lower for less successful projects/programmes. Only nine of the 20 Gavi-supported projects had completed their second year during the period of data collection.

Lessons learnt, drivers of high coverage and key mobilisation messages were consistent across types of demonstration project and world regions, i.e. Africa (23 countries), Asia (10), Americas (7), Oceania (4), Europe (2). Limited EPI involvement is unlikely to be an issue in most future demonstration projects as involvement is required for Gavi applications [10]. Lessons were similar to key findings documented during initial demonstration projects in 2007 [38, 65]. However, the substantial challenges in estimating target population size have not been stressed in previous publications [12, 38]. Enumeration accuracy impacted vaccine requirement projections and coverage calculations in almost all countries included.

Most experience to date is with school-based delivery. Funders should encourage countries to test different approaches as more data is needed on more sustainable strategies. If alternative strategies result in unacceptable levels of coverage, LAMIC may need increased funding to deliver school-based programmes. Limited attempts to reach out-of-school girls did not greatly affect coverage in countries that attain over 80% net school enrolment [14]. However, not providing an opportunity for out-of-school girls to be vaccinated perpetuates inequity.

## Conclusions

HPV vaccine demonstration projects and national programmes to date in LAMICs have achieved high coverage. However, the expense of school-based delivery is of concern for the future sustainability of HPV vaccination programmes. Demonstration projects could better inform national programmes if they provided lessons in challenging areas and populations or tested more sustainable delivery strategies.

## Supporting information

S1 Table An example of the systematic search terms used and results retrieved in the database: Medline (OvidSP); 4^th^ April 2016. (DOCX)

S2 Table Summary of all recommendations. (DOCX)
