# Supplementary material for: Lessons learnt from human papillomavirus (HPV) vaccination in 45 low- and middle-income countries
Source: PLoS One. 2017 Jun 2;12(6):e0177773. doi: 10.1371/journal.pone.0177773 (PMC5456063; doi:10.1371/journal.pone.0177773)
Supplement: S1 Table — (DOCX) [file pone.0177773.s001.docx]

| **Search** |  | **Results** |
| --- | --- | --- |
| 1 | Papillomavirus Vaccines/ | 5085 |
| 2 | hpv.ab,ti. | 26945 |
| 3 | human papillomavirus.ab,ti. | 23071 |
| 4 | human papilloma virus.ab,ti. | 3661 |
| 5 | exp Immunization Programs/ | 10517 |
| 6 | exp Vaccination/ | 70018 |
| 7 | immuni$.ab,ti. | 229073 |
| 8 | vaccin$.ab,ti. | 225394 |
| 9 | 2 or 3 or 4 | 33568 |
| 10 | Immunization/ | 46296 |
| 11 | 5 or 6 or 7 or 8 or 10 | 415268 |
| 12 | 9 and 11 | 7745 |
| 13 | 1 or 12 | 8441 |
| 14 | gambia/ | 2133 |
| 15 | gambia.ab,ti. | 1774 |
| 16 | 14 or 15 | 2621 |
| 17 | limit 16 to yr="2014-Current" | 150 |
| 18 | 13 and 17 | 0 |
| 19 | senegal/ | 4852 |
| 20 | senegal.ab,ti. | 4103 |
| 21 | 19 or 20 | 6035 |
| 22 | limit 21 to yr="2014-Current" | 376 |
| 23 | 13 and 22 | 2 |
| 24 | zimbabwe/ | 4890 |
| 25 | zimbabwe.ab,ti. | 3737 |
| 26 | 24 or 25 | 5778 |
| 27 | limit 26 to yr="2014-Current" | 299 |
| 28 | 13 and 27 | 0 |
| 29 | chile/ | 10403 |
| 30 | chile.ab,ti. | 8360 |
| 31 | 29 or 30 | 12887 |
| 32 | limit 31 to yr="2014-Current" | 990 |
| 33 | 13 and 32 | 6 |
| 34 | burkina faso/ | 2398 |
| 35 | "burkina faso".ab,ti. | 2409 |
| 36 | 34 or 35 | 2980 |
| 37 | limit 36 to yr="2014-Current" | 316 |
| 38 | 13 and 37 | 0 |
| 39 | "cote d'ivoire".ab,ti. | 1431 |
| 40 | cote d'ivoire/ | 2595 |
| 41 | 39 or 40 | 2969 |
| 42 | limit 41 to yr="2014-Current" | 195 |
| 43 | 13 and 42 | 1 |
| 44 | ethiopia/ | 7873 |
| 45 | ethiopia.ab,ti. | 6694 |
| 46 | 44 or 45 | 8911 |
| 47 | limit 46 to yr="2014-Current" | 1268 |
| 48 | 13 and 47 | 1 |
| 49 | "solomon islands".ab,ti. | 508 |
| 50 | solomon islands/ | 892 |
| 51 | 49 or 50 | 1072 |
| 52 | limit 51 to yr="2014-Current" | 62 |
| 53 | 13 and 52 | 0 |
| 54 | togo/ | 870 |
| 55 | togo.ab,ti. | 942 |
| 56 | 54 or 55 | 1134 |
| 57 | limit 56 to yr="2014-Current" | 89 |
| 58 | 13 and 57 | 0 |
| 59 | bhutan/ | 240 |
| 60 | bhutan.ab,ti. | 288 |
| 61 | 59 or 60 | 361 |
| 62 | limit 61 to yr="2009 -Current" | 188 |
| 63 | 13 and 62 | 5 |
| 64 | bolivia/ | 2013 |
| 65 | bolivia.ab,ti. | 2108 |
| 66 | 64 or 65 | 2792 |
| 67 | limit 66 to yr="2009 -Current" | 939 |
| 68 | 13 and 67 | 2 |
| 69 | botswana/ | 1275 |
| 70 | botswana.ab,ti. | 1364 |
| 71 | 69 or 70 | 1648 |
| 72 | limit 71 to yr="2013 -Current" | 283 |
| 73 | 13 and 72 | 3 |
| 74 | brazil/ | 62883 |
| 75 | (brazil or brasil).ab,ti. | 48616 |
| 76 | 74 or 75 | 75353 |
| 77 | limit 76 to yr="2010 -Current" | 29830 |
| 78 | 13 and 77 | 55 |
| 79 | cambodia/ | 2388 |
| 80 | (cambodia or cambodge).ab,ti. | 2169 |
| 81 | 79 or 80 | 3029 |
| 82 | limit 81 to yr="2009 -Current" | 1277 |
| 83 | 13 and 82 | 5 |
| 84 | (cameroon or cameroun).ab,ti. | 4286 |
| 85 | cameroon/ | 4051 |
| 86 | 84 or 85 | 5216 |
| 87 | limit 86 to yr="2010 -Current" | 1691 |
| 88 | 13 and 87 | 7 |
| 89 | georgia/ | 9405 |
| 90 | (georgia or Sakartvelo).ab,ti. | 6753 |
| 91 | 89 or 90 | 12915 |
| 92 | limit 91 to yr="2010 -Current" | 2609 |
| 93 | 13 and 92 | 18 |
| 94 | ghana/ | 5275 |
| 95 | ghana.ab,ti. | 5071 |
| 96 | 94 or 95 | 6357 |
| 97 | limit 96 to yr="2013 -Current" | 1228 |
| 98 | 13 and 97 | 1 |
| 99 | guyana/ | 562 |
| 100 | guyana.ab,ti. | 623 |
| 101 | 99 or 100 | 897 |
| 102 | limit 101 to yr="2012 -Current" | 126 |
| 103 | 13 and 102 | 0 |
| 104 | haiti/ | 2504 |
| 105 | haiti.ab,ti. | 1958 |
| 106 | 104 or 105 | 2948 |
| 107 | limit 106 to yr="2009 -Current" | 1325 |
| 108 | 13 and 107 | 9 |
| 109 | honduras/ | 907 |
| 110 | honduras.ab,ti. | 1125 |
| 111 | 109 or 110 | 1361 |
| 112 | limit 111 to yr="2011 -Current" | 265 |
| 113 | 13 and 112 | 7 |
| 114 | india/ | 82017 |
| 115 | india.ab,ti. | 53099 |
| 116 | 114 or 115 | 96520 |
| 117 | limit 116 to yr="2009 -Current" | 32162 |
| 118 | 13 and 117 | 100 |
| 119 | kenya/ | 12121 |
| 120 | kenya.ab,ti. | 11071 |
| 121 | 119 or 120 | 14645 |
| 122 | limit 121 to yr="2011 -Current" | 3798 |
| 123 | 13 and 122 | 13 |
| 124 | kiribati/ | 1003 |
| 125 | kiribati.ab,ti. | 110 |
| 126 | 124 or 125 | 1059 |
| 127 | limit 126 to yr="2011 -Current" | 163 |
| 128 | 13 and 127 | 0 |
| 129 | (laos or lao).ab,ti. | 2008 |
| 130 | laos/ | 1391 |
| 131 | 129 or 130 | 2431 |
| 132 | limit 131 to yr="2013 -Current" | 404 |
| 133 | 13 and 132 | 0 |
| 134 | lesotho/ | 311 |
| 135 | lesotho.ab,ti. | 422 |
| 136 | 134 or 135 | 481 |
| 137 | limit 136 to yr="2009 -Current" | 157 |
| 138 | 13 and 137 | 1 |
| 139 | madagascar/ | 2581 |
| 140 | madagascar.ab,ti. | 3029 |
| 141 | 139 or 140 | 3543 |
| 142 | limit 141 to yr="2013 -Current" | 549 |
| 143 | 13 and 142 | 0 |
| 144 | malawi/ | 3555 |
| 145 | malawi.ab,ti. | 3743 |
| 146 | 144 or 145 | 4441 |
| 147 | limit 146 to yr="2013 -Current" | 934 |
| 148 | 147 and 13 | 3 |
| 149 | mali/ | 1862 |
| 150 | mali.ab,ti. | 2281 |
| 151 | 149 or 150 | 2754 |
| 152 | limit 151 to yr="2012 -Current" | 569 |
| 153 | 152 and 13 | 5 |
| 154 | (moldova or moldavia).ab,ti. | 516 |
| 155 | moldova/ | 604 |
| 156 | 154 or 155 | 882 |
| 157 | limit 156 to yr="2013 -Current" | 98 |
| 158 | 157 and 13 | 2 |
| 159 | mongolia/ | 1306 |
| 160 | mongolia.ab,ti. | 2126 |
| 161 | 159 or 160 | 2647 |
| 162 | limit 161 to yr="2012 -Current" | 747 |
| 163 | 162 and 13 | 1 |
| 164 | morocco/ | 4302 |
| 165 | morocco.ab,ti. | 3413 |
| 166 | 164 or 165 | 5304 |
| 167 | 13 and 166 | 6 |
| 168 | mozambique/ | 1622 |
| 169 | mozambique.ab,ti. | 1974 |
| 170 | 168 or 169 | 2323 |
| 171 | limit 170 to yr="2014 -Current" | 302 |
| 172 | 171 and 13 | 0 |
| 173 | nepal/ | 5554 |
| 174 | nepal.ab,ti. | 5146 |
| 175 | 173 or 174 | 6611 |
| 176 | limit 175 to yr="2008 -Current" | 3107 |
| 177 | 176 and 13 | 6 |
| 178 | niger/ | 947 |
| 179 | niger.ab,ti. | 8535 |
| 180 | 178 or 179 | 8706 |
| 181 | limit 180 to yr="2014 -Current" | 692 |
| 182 | 181 and 13 | 0 |
| 183 | papua new guinea.ab,ti. | 3512 |
| 184 | papua new guinea/ | 2964 |
| 185 | 183 or 184 | 4294 |
| 186 | limit 185 to yr="2012 -Current" | 570 |
| 187 | 186 and 13 | 1 |
| 188 | peru/ | 6144 |
| 189 | peru.ab,ti. | 6012 |
| 190 | 188 or 189 | 8298 |
| 191 | limit 190 to yr="2007 -Current" | 3606 |
| 192 | 191 and 13 | 26 |
| 193 | (philippines or pilipinas or filipinas).ab,ti. | 5438 |
| 194 | philippines/ | 6935 |
| 195 | 193 or 194 | 8853 |
| 196 | limit 195 to yr="2010 -Current" | 1830 |
| 197 | 196 and 13 | 4 |
| 198 | rwanda/ | 1649 |
| 199 | rwanda.ab,ti. | 1532 |
| 200 | 198 or 199 | 2072 |
| 201 | limit 200 to yr="2011 -Current" | 617 |
| 202 | 201 and 13 | 8 |
| 203 | sierra leone.ab,ti. | 1053 |
| 204 | sierra leone/ | 948 |
| 205 | 203 or 204 | 1290 |
| 206 | limit 205 to yr="2013 -Current" | 377 |
| 207 | 206 and 13 | 0 |
| 208 | south africa.ab,ti. | 19388 |
| 209 | south africa/ | 33165 |
| 210 | 208 or 209 | 37735 |
| 211 | limit 210 to yr="2011 -Current" | 8784 |
| 212 | 211 and 13 | 36 |
| 213 | tanzania/ | 8464 |
| 214 | tanzania.ab,ti. | 7676 |
| 215 | 213 or 214 | 9927 |
| 216 | limit 215 to yr="2010 -Current" | 3322 |
| 217 | 216 and 13 | 17 |
| 218 | thailand/ | 21183 |
| 219 | thailand.ab,ti. | 17744 |
| 220 | 218 or 219 | 26226 |
| 221 | limit 220 to yr="2010 -Current" | 7834 |
| 222 | 221 and 13 | 37 |
| 223 | uganda/ | 8601 |
| 224 | uganda.ab,ti. | 8087 |
| 225 | 223 or 224 | 10265 |
| 226 | limit 225 to yr="2008 -Current" | 4692 |
| 227 | 226 and 13 | 32 |
| 228 | uzbekistan/ | 1804 |
| 229 | uzbekistan.ab,ti. | 856 |
| 230 | 228 or 229 | 1999 |
| 231 | limit 230 to yr="2009 -Current" | 242 |
| 232 | 231 and 13 | 2 |
| 233 | vietnam/ | 9403 |
| 234 | vietnam.ab,ti. | 8707 |
| 235 | 233 or 234 | 12413 |
| 236 | limit 235 to yr="2008 -Current" | 4654 |
| 237 | 236 and 13 | 27 |
| 238 | zambia/ | 3347 |
| 239 | zambia.ab,ti. | 3148 |
| 240 | 238 or 239 | 4135 |
| 241 | limit 240 to yr="2013 -Current" | 640 |
| 242 | 241 and 13 | 3 |
| 243 | 18 or 23 or 28 or 33 or 38 or 43 or 48 or 53 or 58 or 63 or 68 or 73 or 78 or 83 or 88 or 93 or 98 or 103 or 108 or 113 or 118 or 123 or 128 or 133 or 138 or 143 or 148 or 153 or 158 or 163 or 167 or 172 or 177 or 182 or 187 or 192 or 197 or 202 or 207 or 212 or 217 or 222 or 227 or 232 or 237 or 242 | 398 |
| 244 | developing countries/ | 65420 |
| 245 | limit 244 to yr="2007 -Current" | 17905 |
| 246 | 245 and 13 | 142 |
| 247 | GAVI.ab,ti. | 238 |
| 248 | limit 247 to yr="2007 -Current" | 213 |
| 249 | 248 and 13 | 31 |
| 250 | (Low-income countries or LIC).ab,ti. | 3274 |
| 251 | limit 250 to yr="2007 -Current" | 2510 |
| 252 | 251 and 13 | 19 |
| 253 | (Low-middle income countries or LMIC).ab,ti. | 456 |
| 254 | limit 253 to yr="2007 -Current" | 445 |
| 255 | 254 and 13 | 2 |
| 256 | 243 or 246 or 249 or 252 or 255 | 545 |
