# Supplementary material for: Lessons learnt from human papillomavirus (HPV) vaccination in 45 low- and middle-income countries
Source: PLoS One. 2017 Jun 2;12(6):e0177773. doi: 10.1371/journal.pone.0177773 (PMC5456063; doi:10.1371/journal.pone.0177773)
Supplement: S2 Table — (DOCX) [file pone.0177773.s002.docx]

**S2 Table. Summary of all recommendations**

| **Section** | **Recommendations** |
| --- | --- |
| **Preparation** | - Selection of districts for demonstration projects should include some challenging areas or populations. - Planning processes should include representatives from the MOH, MOE, and MOF. EPI involvement is critical for effective vaccine delivery. - HPV vaccine delivery should be coordinated with routine EPI vaccine delivery. - A headcount could be the only accurate way of estimating the target population size in schools, CHWs should be mobilised to enumerate out of school girls. - Adequate training is needed in order that staff understand key messages, eligibility criteria and to ensure that coverage estimates are accurate. - Cascade training can be more efficient and less expensive than centralized training sessions; however, should be monitored and evaluated by national level staff. |
| **Communications** | - Social mobilisation in communities should begin at least one month before vaccination, earlier if possible. Time required (e.g. funds disbursement, printing) should not be underestimated. - Messages should focus on: cervical cancer prevention; safety and efficacy, including lack of fertility impact or long-term adverse effects, government endorsement, delivery timing and venues and the need to return for a second dose. - Consent should be opt-out where feasible, ensuring consistency with existing EPI consent policy. If opt-in consent is chosen for HPV vaccination, processes should be streamlined and reasons clearly explained to parents and communities. An example of a streamlined process might be to implement a school health programme consent form at enrolment for all interventions delivered in schools. |
| **Delivery** | - Countries should select a delivery strategy based on a combination of country specific factors: the proportion of the target group enrolled in school, absenteeism, operational costs, desired/adequate coverage, and programme sustainability. - In areas with variable school attendance, an opportunity for out of school girls to receive the vaccine should be provided. A specific mobilisation strategy for out-of-school girls to encourage them to attend vaccination days at schools, outreach sites or the nearest health centre should be implemented. - The grade based eligibility criteria in a school programme is the easiest and quickest strategy to implement; however, we recommend taking into account country specific factors of acceptability to the target group and school enrolment statistics. Grade based criteria can cause challenges when calculating coverage estimates unless age is also recorded. - If resources allow, planning a two-stage delivery of each dose can be successful in reaching those girls who initially refused vaccination, especially when implementing HPV vaccination for the first time. - Vaccination teams can include teachers and CHWs in order to decrease the number of qualified nurses needed for vaccine delivery sessions. - Integration with other outreach activities, spreading HPV vaccine activities over a longer time period, task shifting to lower cadre staff, and/or allowing for longer working days could minimize the impact of HPV vaccine activities on other routine services if human resources are thought to be limited in country. These strategies and/or other strategies should be tested and evaluated. - Non-EPI stakeholders, such as teachers and parents, should be involved in monitoring and reporting AEs. - Countries need to be aware that HIV infected girls require 3 doses and should develop specific strategies to offer them the 3-dose regimen. - Whilst funding from international partners is available it may be worthwhile to maintain a wide age range of eligibility criteria for the first few years of national programmes e.g. 9-13 year old girls. The first few years of implementation can act as a small catch up campaign; subsequent years would then reduce to a single age cohort of 9 year olds. |
| **Achievements** | - Including a component of school-based delivery can yield high coverage and is recommended if resources allow. If school enrolment is low, a mixture of strategies could be important in order to attain good coverage. - More evaluation of health facility only strategies is needed. - An opportunity for girls who missed doses to receive the vaccine should be supplied, either at return visits to schools or referral to health facility or outreach sites, depending on the resources available. |
| **Sustainability** | - More research should be conducted on scale-up experiences. - Further exploration of sustainable funding alternatives should be conducted and disseminated, to encourage more countries to scale-up demonstration projects. - Where feasible (e.g. in terms of funding and country experience with introducing vaccines), consider phased national implementation rather than demonstration projects. |
| **Integration of HPV vaccine with EPI and the health system** | - Rigorous evaluation of combined interventions with HPV vaccine delivery is needed to assess the effect on implementation, coverage, workload and cost. Funding agencies should systematically encourage this. - Gradual integration of processes into routine processes should be planned and formalised after the first round of vaccination is completed (notably for activities such as communication, reporting procedures and processes, supervision, social mobilisation, remuneration, and human resources management). - Opportunities to initiate or strengthen existing school health programmes and/or pre-adolescent/adolescent health should be seized through on-going collaboration with partners (e.g. MOE, reproductive health departments). - HPV vaccine is overwhelmingly being delivered through “routinized” campaigns, it is critical to ensure that other routine health services are not disrupted by recurrent school delivery or that possible disruptions are mitigated, and that this delivery strategy is sustainably funded. This needs to be monitored and evaluated. |
